# Symptom experience subtypes and their association with social isolation in older adults with diabetes and coronary heart disease: a latent profile analysis study

**DOI:** 10.3389/fmed.2026.1756120

**Published:** 2026-03-02

**Authors:** Niuniu Zhou, Yuzhong Gu, Jianyun Liu, Lingyan Zhang, Ling Chen, Yi Lu, Tingyu Peng

**Affiliations:** 1 Department of Endocrinology, The Sixth People's Hospital of Nantong, Nantong, Jiangsu, China; 2 Department of Cardiology, The Sixth People's Hospital of Nantong, Nantong, Jiangsu, China

**Keywords:** comorbidity, coronary heart disease, diabetes mellitus, latent profile analysis, older adults, social isolation, symptom clusters

## Abstract

**Objective:**

To identify latent classes based on symptom clusters and to explore the association between these distinct symptom experience subtypes and social isolation in older adults with comorbid diabetes mellitus (DM) and coronary heart disease (CHD).

**Methods:**

A cross-sectional study was conducted among 337 older adults with DM and CHD recruited from the Department of Endocrinology and Cardiology of Nantong Sixth People’s Hospital between February 2023 and October 2025. Data were collected using a general information questionnaire, the Chinese version of the Memorial Symptom Assessment Scale (MSAS), and the Lubben Social Network Scale-6 (LSNS-6). Exploratory factor analysis (EFA) was used to identify symptom clusters. Latent profile analysis (LPA) was then employed to classify patients into different symptom experience subtypes based on the symptom cluster scores. One-way ANOVA, Chi-square tests, and multiple linear regression were used to analyze the association between latent classes and social isolation.

**Results:**

EFA extracted three symptom clusters (cardiopulmonary-fatigue, emotional-perceptual, and metabolic), accounting for 62.3% of the total variance. LPA identified three distinct latent classes: Class 1 “Low Burden-Balanced Pattern” (45.4%), Class 2 “Psycho-Somatic Co-dominant Pattern” (31.8%), and Class 3 “Metabolic-Physical Dominant Pattern” (22.8%). Univariate analysis revealed significant differences in social isolation scores (LSNS-6) across the three classes (*F* = 35.67, *p* < 0.001). Multiple linear regression analysis, after adjusting for confounders, indicated that compared to Class 1, both Class 2 (*β* = −4.82, *p* < 0.001) and Class 3 (*β* = −3.25, p < 0.001) were significantly associated with lower LSNS-6 scores, suggesting a greater degree of social isolation.

**Conclusion:**

The findings reveal significant heterogeneity in symptom experiences among older adults with comorbid DM and CHD, which can be categorized into distinct latent classes. The subtype characterized by a Psycho-Somatic Co-dominant Pattern shows the strongest association with social isolation. In clinical practice, early identification of this high-burden subgroup may facilitate the provision of integrated interventions that address physical, psychological, and social dimensions.

## 1 Introduction

Diabetes mellitus (DM) and coronary heart disease (CHD) are common chronic conditions that pose serious threats to the health of older adults. These two diseases frequently coexist, forming a complex comorbid state (1, 2). Against the backdrop of a rapidly aging global population, multimorbidity among older adults has become an increasingly prominent public health challenge. It is estimated that the prevalence of having two or more chronic diseases among individuals aged 65 and older is projected to rise from 54% in 2015 to 68% by 2035 (3). This trend not only signifies a shift in the chronic disease spectrum but also presents severe challenges to healthcare systems worldwide, prompting countries to prioritize comorbidity management as a core issue in healthy aging policies. In this context, an in-depth investigation into the health status of older adults with multimorbidity and its influencing factors is of significant practical importance for formulating targeted prevention and treatment strategies and optimizing resource allocation. Within this context, the number of patients with comorbid DM and CHD is substantial, placing a significant burden on healthcare systems (4). Current management strategies for these patients primarily focus on the stringent control of risk factors such as blood glucose, blood pressure, and blood lipids (5).

However, due to the pathophysiological interactions between the two diseases and the complexities of polypharmacy, patients often experience multiple, concurrent symptoms. Studies indicate that over 80% of older adults with DM and CHD report symptoms such as fatigue and shortness of breath (6, 7), while 30–50% experience pain, sleep disturbances, and exhibit a significantly higher incidence of psychological symptoms like anxiety and depression compared to the general population (8, 9). These symptoms do not occur in isolation but are interrelated and synergistic, often presenting as “symptom clusters.” Failure to address the management of these symptom clusters can significantly increase the patient’s disease burden, adversely affecting treatment adherence and clinical prognosis (10).

Social isolation is a critical psychosocial factor influencing health in older age, closely associated with disability, cognitive decline, and increased mortality (11). For older patients with DM and CHD, a high symptom burden, particularly from complex symptom clusters, may exacerbate the risk of social isolation through various pathways. These include limiting physical mobility, triggering negative emotions, and increasing the burden of disease management, which can impede social participation (12, 13).

Nevertheless, existing research has predominantly focused on the relationship between single symptoms or total symptom scores and social support, overlooking the heterogeneity within the patient population (14). Different patients may experience distinct symptom cluster patterns, and the strength of the association between these specific patterns and social isolation may vary. Currently, studies on symptom clusters in older adults with multimorbidity are largely descriptive, lacking a shift from a “variable-centered” to a “person-centered” perspective. Latent profile analysis (LPA), an advanced statistical method, can categorize individuals into mutually exclusive subgroups based on their external characteristics (e.g., symptom cluster profiles), thereby identifying “patient subtypes” with similar symptom experiences (15). This classification method helps uncover population heterogeneity and provides targets for precise interventions.

Therefore, this study innovatively integrates the concepts of “symptom clusters” and “latent profile analysis.” Through a cross-sectional survey, it aims to: first, identify the structure of symptom clusters in older adults with comorbid DM and CHD; second, explore potential classes of their symptom experiences; and third, thoroughly analyze the association between different symptom experience subtypes and the degree of social isolation. The findings will assist clinicians in early identification of patient subtypes at high risk for social isolation, providing crucial evidence for developing precise symptom cluster management and psychosocial intervention strategies. Ultimately, this research aims to improve patients’ quality of life and long-term prognosis.

## 2 Objects and methods

### 2.1 Study objects

This study utilized a convenience sampling approach to enroll participants from the Departments of Endocrinology and Cardiology at Nantong Sixth People’s Hospital between February 2023 and October 2025. The study cohort consisted of older adult patients diagnosed with comorbid diabetes mellitus and coronary heart disease. Inclusion criteria were rigorously defined as follows: 1) Fulfillment of the diagnostic standards specified in the Chinese Guidelines for the Prevention and Treatment of Type 2 Diabetes (2013 Edition) (16) and the Diagnostic Criteria for Coronary Heart Disease (17); 2) Attainment of age 60 years or older; 3) Full consciousness without cognitive impairment or communication barriers, coupled with fundamental literacy skills; 4) Provision of informed consent with voluntary participation agreement. Exclusion criteria encompassed: 1) Presence of severe comorbidities (e.g., malignancies, significant hepatic or renal dysfunction, advanced heart failure) or acute complications (e.g., diabetic ketoacidosis, serious infections); 2) Documented history of psychiatric disorders or cognitive impairments (e.g., Alzheimer’s disease). Withdrawal criteria included: Participant-initiated revocation of informed consent during the investigation process or health deterioration preventing questionnaire completion.

Sample size determination followed methodological requirements for exploratory factor analysis. According to Kendall’s fundamental principle (18), the sample size should generally range from 5 to 10 times the number of variables. With 32 symptoms assessed through the Memorial Symptom Assessment Scale, the initial minimum sample was calculated as 32 × 5 = 160 participants. Further refined by Comrey and Lee’s classification framework (19)—where 50 represents “very poor,” 100 “medium,” 200 “good,” 300 “very good,” and 500 “excellent”—we established a target sample size exceeding the “very good” threshold (300 participants) to ensure robust analytical reliability. Accounting for latent profile analysis requirements and an estimated 10% invalid response rate, the final target was set at 337 participants. The investigation successfully collected 350 questionnaires, and after excluding 13 invalid responses due to incomplete or patterned answers, the final analytical sample comprised 337 cases, satisfying all methodological requirements.

The study protocol received full approval from the Ethics Committee of Nantong Sixth People’s Hospital (Approval No.: NTLYLL2023003; Approval Date: January 7, 2023). All investigative procedures were conducted in strict adherence to ethical guidelines and regulatory standards.

### 2.2 Survey instruments

#### 2.2.1 General information questionnaire

A self-administered general information questionnaire was designed for this study. It comprised two main sections: Sociodemographic Data: This section collected information on patient gender, age, educational attainment, marital status, living arrangements, and Body Mass Index (BMI).

Disease-related clinical data: This section included information on diabetes type, duration of diabetes, glycemic control indicators (such as glycated hemoglobin HbA1c), duration of CHD, cardiac function classification (NYHA), and comorbidities (e.g., hypertension, hyperlipidemia, chronic kidney disease). To ensure data accuracy, all disease-related clinical information was verified and completed by the investigators through reviewing patients’ electronic medical records and the Hospital Information System (HIS).

#### 2.2.2 Symptom assessment tool

The Chinese version of the Memorial Symptom Assessment Scale (MSAS) was employed to evaluate patients’ symptom experiences (20). This scale was developed by Portenoy et al. (21) and is widely used internationally as a tool for assessing the multi-dimensional symptom experience of patients with chronic diseases. This instrument is internationally recognized for assessing multidimensional symptom experiences in patients with chronic conditions and demonstrates good reliability and validity (22). The scale encompasses 32 core symptom items, comprehensively covering both physical and psychological dimensions. Furthermore, it includes one open-ended question, allowing patients to supplement other significant symptoms they experienced but were not listed, thereby ensuring assessment comprehensiveness. Each symptom was evaluated across four dimensions: Occurrence: Assessed whether the symptom was present in the past week (Yes/No). Severity: If the symptom occurred, its severity was rated on a 4-point scale (1 = Mild, 2 = Moderate, 3 = Severe, 4 = Very severe). Frequency: If the symptom occurred, its frequency was rated on a 4-point scale (1 = Rarely, 2 = Occasionally, 3 = Frequently, 4 = Almost constantly). Distress: If the symptom occurred, the subjective distress it caused to the patient’s emotions and daily life was rated on a 5-point scale (0 = Not at all, 1 = A little bit, 2 = Somewhat, 3 = Quite a bit, 4 = Very much).

Application and scoring in this study: As this study aimed to identify symptom clusters and patient subtypes, primary emphasis was placed on the “Distress” dimension scores. This dimension most effectively reflects the symptom’s subjective impact on overall patient functioning and wellbeing, serving as a core indicator linking physiological sensation to psychosocial outcomes such as social isolation. Consequently, for symptom cluster analysis, the distress rating (0–4) for each symptom was utilized as the primary analytical variable. Symptoms that did not occur were assigned a distress score of 0.

Symptom cluster identification method: Following data preprocessing, the distress scores for the 32 symptoms were subjected to exploratory factor analysis (EFA) to identify clusters of symptoms with strong internal correlations, potentially sharing common pathophysiological mechanisms (i.e., symptom clusters). Principal component analysis with varimax rotation was applied. Factors were extracted based on eigenvalues greater than 1.0 and factor loadings exceeding 0.4. Each extracted factor represented a distinct symptom cluster. The mean distress score of all symptoms loading significantly onto a factor was calculated for each patient, representing their score for that specific symptom cluster. These cluster scores were subsequently used for the latent profile analysis. The Chinese version of the MSAS demonstrated good internal consistency in our study population, with a Cronbach’s *α* coefficient ranging from 0.79 to 0.87, indicating reliable measurement outcomes.

#### 2.2.3 Lubben Social Network Scale-6 (LSNS-6)

This scale was developed by Lubben et al. (23) and is designed to briefly assess the strength of social support networks among the olderly, with a focus on the size, frequency of contact, and closeness of family and friend relationships. This instrument is a validated tool designed to evaluate an individual’s social network size across two key dimensions: family networks (3 items) and friend networks (3 items). Each item on the LSNS-6 is scored on a 6-point scale, ranging from 0 (indicating none) to 5 (indicating all). The total possible score sums to a maximum of 30 points, providing a composite measure of overall social connectivity. The interpretation of the scoring results is conducted as follows: The total score serves as a primary indicator of overall risk for social isolation. A total score of less than 12 points is classified as indicative of a state of social isolation. Furthermore, subscale scores for the family and friend dimensions offer insights into specific network deficiencies. A score below 6 points on either the family or friend subscale suggests the presence of isolation within that particular relational domain, signifying either family isolation or friend isolation, respectively. The internal consistency reliability of the LSNS-6 was confirmed within the context of this specific study population. The analysis demonstrated good reliability, with a Cronbach’s alpha coefficient of 0.800 for the family network subscale and 0.890 for the friend network subscale, indicating that the scale performed consistently in measuring these constructs among the participants.

### 2.3 Data collection methods

This study adopted a cross-sectional survey design, implementing a standardized data collection protocol to ensure the accuracy and consistency of all gathered information. The specific procedures were executed as follows: First, all members of the research team received unified training on the study protocol before commencing data collection. The process began with investigators conducting daily screenings of the Hospital Information System (HIS). Potential participants were initially identified from among inpatients in the Endocrinology and Cardiology departments based on the predefined inclusion and exclusion criteria. Subsequently, after securing approval from the attending physicians, investigators approached the potential participants in the wards. Each eligible patient was provided with a comprehensive explanation regarding the study’s purpose, significance, detailed content, and the estimated time commitment. Their rights as participants were explicitly communicated. Following this, written informed consent was obtained from each patient after ensuring their full understanding of the study details. Data collection was then conducted in a quiet office or treatment room within the ward to ensure privacy and minimize distractions. The survey was administered using a “one-on-one, face-to-face” interview technique. In this method, the investigator read each questionnaire item aloud to the participant, who then provided answers based on their personal experiences and current condition. For older patients with lower educational attainment or visual impairments, the investigator took special care to ensure the patient fully comprehended each question’s meaning before recording the responses on their behalf. All questionnaires were collected on-site immediately upon completion. The investigators performed preliminary checks to assess the completeness and logical consistency of the responses. Any instances of missing data or contradictory answers were promptly addressed by verifying the information directly with the patient to ensure data integrity before the conclusion of the session. This rigorous process was implemented to maintain high-quality data standards throughout the study.

### 2.4 Statistical methods

Data analysis was performed using SPSS version 26.0 and Mplus version 8.3 statistical software. The presentation of data adhered to standard statistical conventions: continuous variables were described using mean ± standard deviation if normally distributed, or median (interquartile range) if non-normally distributed. Categorical data were summarized as frequencies and percentages. The significance level (*α*) was set at 0.05 for two-tailed tests.

Data preprocessing: A comprehensive logical check was first conducted on the entire dataset. An analysis of missing data for the MSAS and LSNS-6 items revealed high data completeness, with rates of 99.2% for the 32 symptom items and 99.8% for the 6 LSNS-6 items. Given the very low overall missing rate (<1%) and Little’s Missing Completely at Random (MCAR) test indicating the data were missing completely at random (*p* > 0.05), the mean imputation method was employed to handle the minimal missing values in individual items. This approach was chosen to preserve the sample size and maintain statistical power. All subsequent analyses were based on this complete dataset.

Identification of symptom clusters: Exploratory factor analysis (EFA) was conducted on the “distress” ratings of the 32 symptoms from the MSAS to identify symptom clusters. The principal component analysis method was used for factor extraction, coupled with varimax rotation. The criteria for determining the number and structure of the factors (symptom clusters) included an eigenvalue greater than 1.0 and a factor loading absolute value exceeding 0.40. For each resulting symptom cluster, a mean distress score was calculated for each patient by averaging the distress ratings of all symptoms loading significantly onto that factor. This cluster score was used in subsequent analyses.

Identification of latent classes of symptom experience: LPA was performed using the factor scores derived from the EFA (i.e., the symptom cluster scores) as continuous indicator variables. Models ranging from 1 to 5 latent classes were estimated and compared. The optimal number of classes was determined by evaluating multiple fit indices: lower values on the Akaike information criterion (AIC) and Bayesian information criterion (BIC) indicated better model fit; an entropy value greater than 0.80 suggested high classification accuracy; and a significant *p*-value (*p* < 0.05) for the Lo–Mendell–Rubin adjusted Likelihood Ratio Test (LMRT) and the Bootstrap Likelihood Ratio Test (BLRT) indicated that the K-class model provided a statistically superior fit to the (K-1)-class model. The final model selection integrated statistical fit indices with considerations of model parsimony, class probabilities, and clinical interpretability.

Group comparisons and analysis of association with social isolation: Univariate analyses were conducted to examine differences in social isolation across the identified latent classes. One-way analysis of variance (ANOVA) or the Kruskal–Wallis *H* test was used to compare differences in the continuous social isolation total score (LSNS-6 total) among the latent classes. The Chi-square test was employed to compare the proportions of patients categorized as socially isolated (using the standard cutoff of LSNS-6 total score < 12) across the different latent classes. To control for potential confounding effects, a multiple linear regression analysis was performed. In this model, the social isolation total score served as the dependent variable, and the latent class membership was the primary independent variable (using dummy coding). Covariates included in the model were variables that showed statistical significance in the univariate analyses or were considered clinically important. The results of the regression analysis are presented as unstandardized regression coefficients (*β*) along with their 95% confidence intervals.

## 3 Results

### 3.1 Baseline characteristics of the study participants

This study successfully included a total of 337 valid samples for final analysis. The detailed sociodemographic and clinical characteristics of the study participants are comprehensively summarized in Table 1. As clearly demonstrated in the table, the age of the enrolled patients ranged from 60 to 89 years, with a mean age of (71.25 ± 7.2) years. In terms of gender distribution, the cohort comprised 198 male patients, accounting for 58.8% of the total sample, and 139 female patients, representing the remaining 41.2%. This indicates a relatively higher representation of males within the study population.

**Table 1:** Sociodemographic and clinical characteristics of the study participants (*n* = 337).

Characteristic	Value/frequency	Proportion (%)/range
Age (years), mean ± SD	71.5 ± 7.2	60–89
Gender, *n* (%)		
Male	198	58.8
Female	139	41.2
Marital status (married/cohabiting), *n* (%)	265	78.6
Educational level, *n* (%)		
Primary school or below	145	43.0
Middle school/technical secondary school	127	37.7
College or above	65	19.3
BMI (kg/m^2^), mean ± SD	25.6 ± 3.4	
BMI category (Chinese criteria), *n* (%)		
Normal (<24 kg/m^2^)	118	35.0
Overweight (24–27.9 kg/m^2^)	156	46.3
Obesity (≥28 kg/m^2^)	63	18.7
Diabetes duration (years), median (Q1, Q3)	9.0 (5.0, 14.0)	1–28
CHD duration (years), median (Q1, Q3)	5.0 (2.0, 8.0)	1–22
Glycated hemoglobin (HbA1c) (%), mean ± SD	7.8 ± 1.5	5.2–12.5
Number of comorbidities, mean ± SD	2.8 ± 1.3	1–6
Major comorbidities, *n* (%)		
Hypertension	295	87.5
Hyperlipidemia	226	67.1
Chronic kidney disease	91	27.0
NYHA Heart Function Classification, *n* (%)		
Class I	85	25.2
Class II	185	54.9
Class III	62	18.4
Class IV	5	1.5

BMI, Body Mass Index; NYHA, New York Heart Association.

### 3.2 Identification of symptom clusters in older adult patients with diabetes and coronary heart disease

To identify the underlying structure of symptom clusters, we performed an EFA on the distress ratings of 32 symptoms from the Memorial Symptom Assessment Scale. The data demonstrated excellent suitability for factor analysis, as evidenced by a Kaiser–Meyer–Olkin (KMO) measure of 0.87 (exceeding the 0.80 threshold for adequacy) and a highly significant Bartlett’s test of sphericity (*χ*^2^ = 5123.65, degrees of freedom = 496, *p* < 0.001), indicating substantial correlations among the variables.

**Figure 1:**
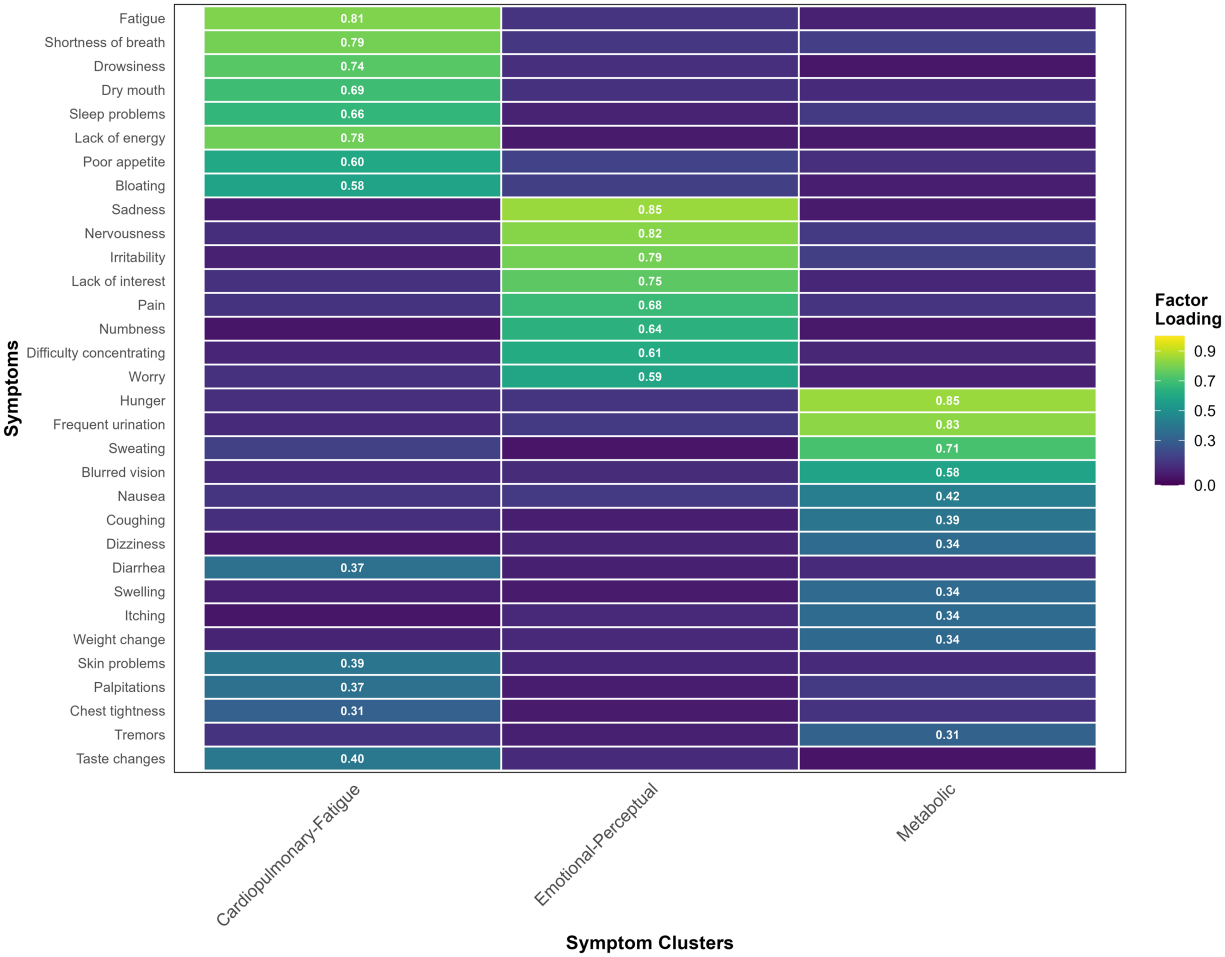
Heatmap of factor loadings for symptoms in older adults with comorbid diabetes and coronary heart disease. The color of each cell represents the factor loading of a specific symptom onto its respective cluster. Warmer colors (yellow) indicate higher loadings (stronger associations), while cooler colors (blue) indicate lower loadings.

Factor extraction and overall model Fit: Principal component analysis with varimax rotation was employed, retaining factors with eigenvalues greater than 1.0. The analysis yielded a three-factor solution, which collectively accounted for 62.3% of the total variance, suggesting a satisfactory representation of the symptom data structure. The factor loadings for each symptom across the three factors are presented in Table 2 and Figure 1. Symptoms with absolute factor loadings exceeding 0.40 were considered meaningful contributors to a specific factor.

**Table 2:** Exploratory factor analysis of symptom clusters in older adults with diabetes and coronary heart disease (*n* = 337).

Symptom	Factor loadings		
	Factor 1: Cardiopulmonary-Fatigue Cluster	Factor 2: Emotional-Perceptual Cluster	Factor 3: Metabolic Cluster
Fatigue	**0.812**	0.152	0.098
Shortness of breath	**0.796**	0.210	0.075
Lack of energy	**0.785**	0.225	0.110
Drowsiness	**0.745**	0.105	0.233
Dry mouth	**0.698**	0.098	0.305
Sleep problems	**0.665**	0.350	0.087
Poor appetite	**0.601**	0.280	0.150
Bloating	**0.580**	0.190	0.210
Sadness	0.118	**0.850**	0.092
Nervousness	0.205	**0.823**	0.110
Irritability	0.178	**0.791**	0.145
Lack of interest	0.250	**0.752**	0.098
Pain	0.320	**0.680**	0.098
Numbness	0.295	**0.642**	0.105
Difficulty concentrating	0.330	**0.615**	0.205
Worrying	0.285	**0.590**	0.185
Hunger	0.150	0.088	**0.855**
Frequent urination	0.208	0.102	**0.830**
Sweating	0.310	0.215	**0.715**
Blurred vision	0.255	0.305	**0.585**
Eigenvalue	5.82	4.12	3.47
Variance explained (%)	26.4	20.1	15.8
Cumulative variance explained (%)	26.4	46.5	62.3

Extraction method: principal component analysis; rotation method: varimax with Kaiser normalization.

Factor loadings with an absolute value > 0.40 are displayed in bold, indicating the primary factor affiliation of the symptom.

Symptoms with factor loadings consistently below 0.40 across all factors (e.g., ‘Diarrhea’ 0.35, ‘Dizziness’ 0.32) are omitted from the table for clarity of the primary cluster structure.

Based on the clinical characteristics of the symptoms loading highly on each factor, the three clusters were named as follows: Factor 1: Cardiopulmonary-Fatigue Cluster (eigenvalue = 5.82; 26.4% variance explained): This cluster primarily encompassed core symptoms such as fatigue, shortness of breath, drowsiness, dry mouth, and sleep difficulties, indicating severe challenges related to energy, respiration, and fundamental physiological functions.

Factor 2: Emotional-Perceptual Cluster (eigenvalue = 4.12; 20.1% variance explained): This cluster was predominantly defined by symptoms like sadness, nervousness, irritability, pain, and numbness, highlighting the significant emotional distress and somatic discomfort perception experienced by patients.

Factor 3: Metabolic Cluster (eigenvalue = 3.47; 15.8% variance explained): This cluster included symptoms such as hunger, frequent urination, sweating, and blurred vision, which are closely associated with the pathophysiological processes of diabetes mellitus.

This three-cluster solution provides a structured framework for understanding the complex symptom experiences in this patient population, moving beyond individual symptoms to identifiable and clinically meaningful groupings.

### 3.3 Latent classes of symptom experience in older adults with diabetes and coronary heart disease

To identify distinct patient subtypes based on their symptom experience patterns, LPA was conducted using the scores from the three symptom clusters derived from the exploratory factor analysis. Models ranging from one to five latent classes were sequentially estimated and compared. The fit indices for these models are detailed in Table 3. The AIC, BIC, and aBIC values generally decreased as the number of classes increased. However, the LMRT for the 4-class and 5-class models was no longer statistically significant, indicating that these more complex models did not provide a statistically superior fit compared to the simpler models. After comprehensive evaluation, the 3-class model was selected as optimal. This model demonstrated lower AIC, BIC, and aBIC values, the highest entropy value, and a statistically significant LMRT result, collectively indicating a model with high classification accuracy, parsimony, and interpretability.

**Table 3:** Comparison of fit indices for latent class models of symptom experience (*n* = 337).

Number of classes	AIC	BIC	aBIC	Entropy	LMRT (*p*-value)	BLRT (*p*-value)	Class probabilities (%)
1-Class	2856.32	2880.15	2860.21	—	—	—	100
2-Class	2634.78	2671.24	2642.89	0.856	<0.001	<0.001	65/35
3-Class	2512.45	2561.54	2524.7	0.912	0.012	<0.001	45/32/23
4-Class	2498.11	2559.83	2514.66	0.894	0.065	<0.001	40/28/20/12
5-Class	2489.95	2564.30	2510.72	0.883	0.124	<0.001	35/25/18/12/10

AIC, Akaike information criterion; BIC: Bayesian information criterion; aBIC, sample-size adjusted BIC; LMRT, Lo–Mendell–Rubin Likelihood Ratio Test; BLRT, Bootstrapped Likelihood Ratio Test.

### 3.4 Naming of latent classes of symptom experience

Based on latent profile analysis (LPA), three distinct latent classes of symptom experience were identified among older adult patients with comorbid diabetes and coronary heart disease. To clearly present the intrinsic characteristics of each class, Table 4 provides a centralized description and comparison of their scores on the core symptom clusters.

**Table 4:** Comparison of symptom cluster scores among different latent classes of symptom experience (*n* = 337).

Latent class	Cardiorespiratory-fatigue symptom cluster score (M ± SD)	Emotional-perceptual symptom cluster score (M ± SD)	Metabolic-related symptom cluster score (M ± SD)	Core characteristics of the class
Class 1 (*n* = 153, 45.4%)	1.9 ± 0.4	2.0 ± 0.5	1.8 ± 0.3	Low and relatively balanced burden across all dimensions.
Class 2 (*n* = 107, 31.8%)	3.1 ± 0.7	3.6 ± 0.8	2.5 ± 0.6	Emotional-perceptual burden is particularly prominent, followed by physical burden.
Class 3 (*n* = 77, 22.8%)	3.5 ± 0.7	2.6 ± 0.5	3.8 ± 0.9	Metabolic burden is the highest, accompanied by significant physical burden.

M ± SD = mean ± standard deviation. This table aims to visually present the characteristics of symptom experience across different latent classes using descriptive statistics.

**Figure 2:**
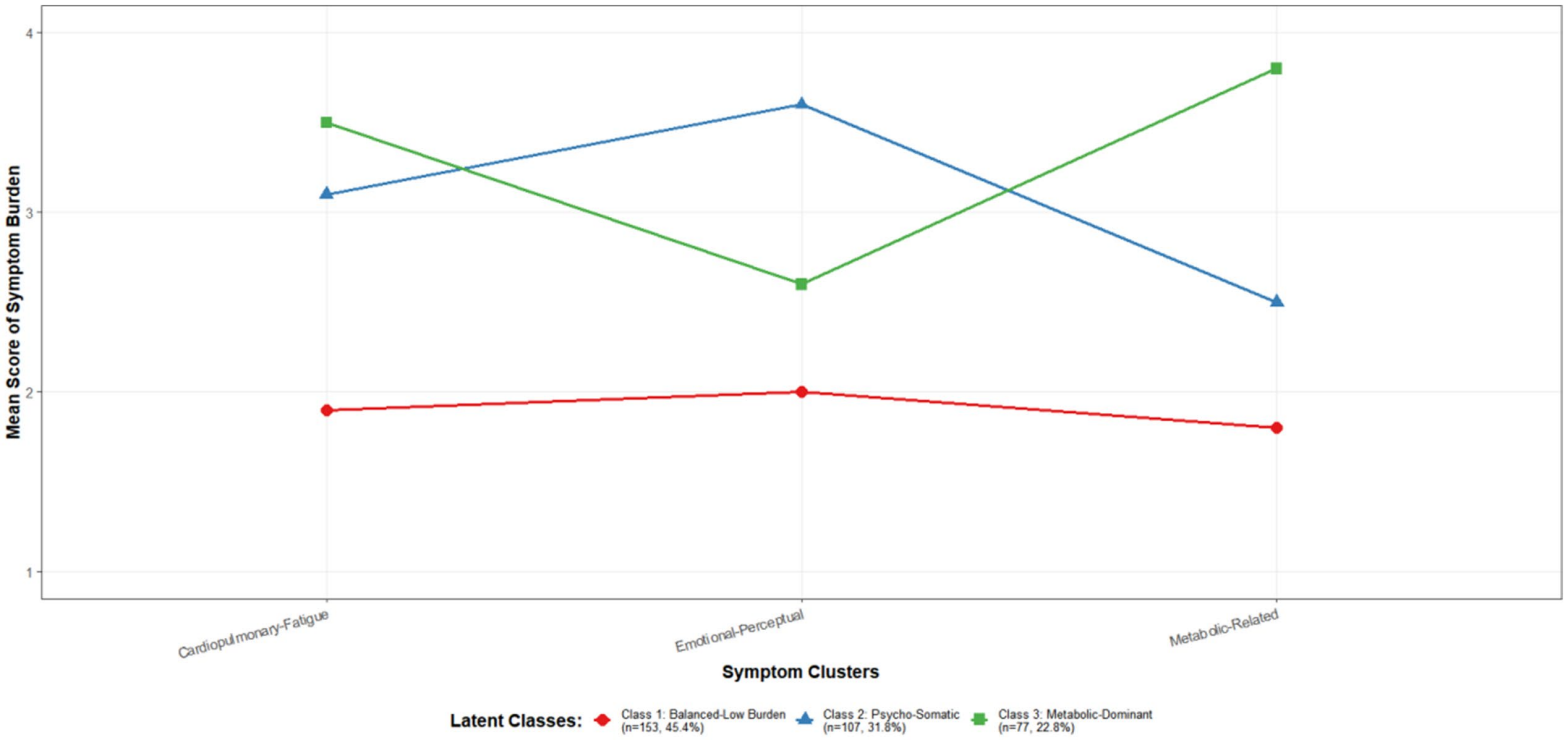
Latent profile of symptom experience in elderly patients with *diabetes mellitus* complicated with coronary heart disease (*n* = 337). Each line represents a latent class. Its profile shape illustrates the class’s unique symptom experience pattern. A higher score indicates a greater degree of distress caused by that symptom cluster.

The mean scores of these three latent classes across the symptom clusters are shown in Figure 2. Their characteristics are described and named as follows: Class 1 (Low Burden-Balanced Pattern): The profile line for this class is relatively flat and positioned low. Their scores on the cardiorespiratory-fatigue (1.9 ± 0.4), emotional-perceptual (2.0 ± 0.5), and metabolic-related (1.8 ± 0.3) symptom clusters are all at a relatively low level, with minimal differences among the three clusters. This indicates that patients in this subgroup experience a mild overall symptom burden with a relatively balanced degree of distress across different dimensions, representing the most clinically stable subgroup. Class 2 (Psycho-Somatic Co-dominant Pattern): The profile line for this class exhibits a typical “psycho-somatic co-occurrence” pattern. Their score on the emotional-perceptual symptom cluster (3.6 ± 0.8) is the highest, while they also demonstrate a high burden on the Cardiorespiratory-Fatigue symptom cluster (3.1 ± 0.7). In contrast, their burden from Metabolic-Related symptoms (2.5 ± 0.6) is relatively lower. This suggests that the core challenge for patients in this class lies in the close intertwining of emotional distress and physical symptoms.

Class 3 (Metabolic-Physical Dominant Pattern): The profile pattern for this class is distinctly different from the previous two. Their score on the Metabolic-Related symptom cluster (3.8 ± 0.9) is the highest, accompanied by a high level of physical burden (3.5 ± 0.7). However, their emotional-perceptual burden (2.6 ± 0.5) is relatively less prominent. This indicates that the symptom experience of patients in this class is primarily driven by metabolic disturbances and the physical fatigue directly induced by them.

### 3.5 Sensitivity analysis

To verify the robustness of the latent class classification results based on symptom cluster scores and to assess the potential impact of factor score uncertainty on the final classification, an additional sensitivity analysis was conducted. This analysis did not rely on the symptom cluster scores generated by Exploratory Factor Analysis. Instead, the “distress” ratings of the 32 symptoms from the MSAS were directly included in the LPA as continuous manifest variables. This analysis aimed to directly investigate whether the data itself, without relying on factor analysis for dimensionality reduction and using all original symptom information, still supported a similar latent class structure.

The model fitting and comparison process for the sensitivity analysis remained consistent with the primary analysis. The model fit indices (see Table 5) indicated that, consistent with the primary analysis, the three-class model was again identified as the optimal model (highest entropy = 0.905, significant LMRT test, and lower information criteria). This result strongly suggests that the latent class structure present in the data is stable and reliable, independent of whether factor analysis is performed first for dimensionality reduction. The symptom experience characteristics of the three latent classes identified in the sensitivity analysis were highly consistent with the results of the primary analysis, further confirming the stable existence of the three patient subtypes: the “Low Burden-Balanced Pattern,” the “Psycho-Somatic Co-dominant Pattern,” and the “Metabolic-Physical Dominant Pattern.”

**Table 5:** Model fit indices for latent profile analysis based on raw symptoms (*n* = 337).

Number of classes	AIC	BIC	aBIC	Entropy	LMRT (*p*-value)	BLRT (*p*-value)	Class probabilities (%)
1-Class	15236.32	15380.15	15280.21	—	—	—	100
2-Class	14834.78	15171.24	14942.89	0.856	<0.001	<0.001	65/35
3-Class	14612.45	15061.54	14724.78	0.905	0.012	<0.001	45/32/23
4-Class	14598.11	15159.83	14714.66	0.884	0.068	<0.001	40/28/20/12
5-Class	14589.95	15264.30	14710.72	0.873	0.130	<0.001	35/25/18/12/10

AIC, Akaike information criterion; BIC, Bayesian information criterion; aBIC: sample-size adjusted BIC; LMRT, Lo–Mendell–Rubin Likelihood Ratio Test; BLRT, Bootstrapped Likelihood Ratio Test.

To quantify the agreement between the two classification methods, a cross-tabulation analysis was performed (see Table 6). The results showed that 92.6% of the samples were assigned to the same class. Cohen’s Kappa coefficient was 0.887 (*p* < 0.001), indicating a high level of agreement between the primary and sensitivity analyses. This fully demonstrates that the core findings of this study regarding the latent classes of symptom experience are highly robust.

**Table 6:** Agreement test between primary analysis and sensitivity analysis classification results (*n* = 337).

Sensitivity analysis (LPA based on raw symptoms)	Primary analysis (LPA based on symptom cluster scores)			Row total
Class A	**142**	8	3	153
Class B	5	**101**	4	110
Class C	2	6	**66**	74
Column total	149	115	73	337

The bold numbers on the main diagonal indicate the number of samples assigned to the same class by both methods. Cohen’s Kappa coefficient = 0.887, *p* < 0.001.

### 3.6 Univariate analysis of social isolation across latent classes of symptom experience

A preliminary univariate analysis was conducted to explore the differences in social isolation levels among patients with different latent classes of symptom experience. As presented in Table 7, significant differences were observed across the three latent classes in terms of the total social isolation score and its subdomain scores (*p* < 0.001).

**Table 7:** Univariate analysis of social isolation across latent classes of symptom experience (*n* = 337).

Variable	Overall (*n* = 337)	Class1 (*n* = 153, 45.4%)	Class 2 (*n* = 107, 31.8%)	Class 3 (*n* = 77, 22.8%)	Statistic	*p*-value
LSNS-6 total score, M ± SD	15.2 ± 5.8	18.5 ± 4.1	12.1 ± 4.9	13.8 ± 5.3	*F* = 35.67	0.001
Family network score, M ± SD	7.8 ± 3.2	9.2 ± 2.5	5.9 ± 2.8	7.1 ± 3.0	*F* = 28.45	<0.001
Friend network score, M ± SD	7.4 ± 3.1	9.3 ± 2.8	6.2 ± 2.7	6.7 ± 2.9	*F* = 31.92	<0.001
Social isolation status, *n* (%)					*χ*^2^ = 40.18	<0.001
Yes (total score < 12)	98 (29.1)	15 (9.8)	55 (51.4)	28 (36.4)		
No (total score ≥ 12)	239 (70.9)	138 (90.2)	52 (48.6)	49 (63.6)		
Family isolation, *n* (%)					*χ*^2^ = 32.75	<0.001
Yes (score < 6)	112 (33.2)	25 (16.3)	62 (57.9)	25 (32.5)		
No (score ≥ 6)	225 (66.8)	128 (83.7)	45 (42.1)	52 (67.5)		
Friend isolation, *n* (%)					*χ*^2^ = 36.44	<0.001
Yes (score < 6)	125 (37.1)	28 (18.3)	68 (63.6)	29 (37.7)		
No (score ≥ 6)	212 (62.9)	125 (81.7)	39 (36.4)	48 (62.3)		

LSNS-6, Lubben Social Network Scale-6; M ± SD, mean ± standard deviation; statistic: *F*-value (one-way ANOVA) for continuous variables, *χ*^2^ value (Chi-square test) for categorical variables. All *p*-values are two-sided. Class 1: Low Burden-Balanced Pattern; Class 2: Psycho-Somatic Co-dominant Pattern; Class 3: Metabolic-Physical Dominant Pattern.

*Post-hoc* pairwise comparisons with Bonferroni correction (Table 8) revealed specific patterns. For the total LSNS-6 score, the mean score for Class 1 (Low Symptom Burden) was significantly higher than that for both Class 2 (mean difference = 6.40, *p* < 0.001) and Class 3 (mean difference = 4.70, *p* < 0.001). Furthermore, Class 2 had a significantly lower mean score than Class 3 (mean difference = −1.70, *p* = 0.048). Regarding the family network subscore, significant differences existed between all three pairs of classes (all *p* < 0.05), with Class 1 exhibiting the highest score and Class 2 the lowest. For the friend network subscore, Class 1 scored significantly higher than both Class 2 (mean difference = 3.10, *p* < 0.001) and Class 3 (mean difference = 2.60, *p* < 0.001); however, no significant difference was found between Class 2 and Class 3 (mean difference = −0.50, *p* = 0.405).

**Table 8:** *Post-hoc* pairwise comparisons of social isolation across latent classes (Bonferroni correction).

Dependent variable	Dependent variable	Mean difference (I–J)	Std. error	*p*-value	95% confidence interval
LSNS-6 total score	Class 1 vs. Class 2	6.40	0.65	<0.001	(5.12, 7.68)
	Class 1 vs. Class 3	4.70	0.70	<0.001	(3.32, 6.08)
	Class 2 vs. Class 3	−1.70	0.75	0.048	(−3.12, −0.28)
Family network score	Class 1 vs. Class 2	3.30	0.35	<0.001	(2.61, 3.99)
	Class 1 vs. Class 3	2.10	0.38	<0.001	(1.35, 2.85)
	Class 2 vs. Class 3	−1.20	0.41	0.011	(−2.02, −0.38)
Friend network score	Class 1 vs. Class 2	3.10	0.34	<0.001	(2.43, 3.77)
	Class 1 vs. Class 3	2.60	0.37	<0.001	(1.87, 3.33)
	Class 2 vs. Class 3	−0.50	0.39	0.405	(−1.28, 0.28)

LSNS-6, Lubben Social Network Scale-6; mean difference = Mean of first group minus mean of second group; Bonferroni correction was applied to control for Type I error in multiple comparisons. Class 1: Low Burden-Balanced Pattern; Class 2: Psycho-Somatic Co-dominant Pattern; Class 3: Metabolic-Physical Dominant Pattern.

The detection rate of social isolation status (LSNS-6 total score < 12) also varied significantly. Class 2 had the highest prevalence rate (51.4%), which was significantly greater than that of Class 1 (9.8%, *p* < 0.001) and Class 3 (36.4%, *p* = 0.021). The prevalence rate for Class 3 was also significantly higher than that for Class 1 (*p* < 0.001).

The univariate analysis results indicate a significant association between latent classes of symptom experience and the degree of social isolation. The patient subtype characterized by high physical-psychological burden (Class 2) experienced the most severe social isolation, particularly within the family network dimension, while patients with low symptom burden (Class 1) had the lowest risk of social isolation.

### 3.7 Multivariate regression analysis of the association between symptom experience latent classes and social isolation

#### 3.7.1 Variable selection and model specification

To examine the independent association between symptom experience latent classes and social isolation while comprehensively controlling for potential confounding factors, this study constructed a fully adjusted multiple linear regression model. The total score of the LSNS-6 served as the dependent variable. The independent variable was the latent class of symptom experience, with “Class 1: Low Burden-Balanced Pattern” designated as the reference category. Covariate selection was based a priori on prior literature and clinical relevance, not on statistical significance from univariate analyses. Accordingly, the following variables were included as covariates: age, sex, education level, marital status, duration of diabetes, duration of coronary heart disease, number of comorbidities, and glycated hemoglobin (HbA1c) level. The univariate association results presented in Table 9 are for descriptive purposes only and were not used for variable selection. All pre-specified covariates were forced into the final regression model to ensure sufficient adjustment and to avoid bias from data-driven variable selection.

**Table 9:** Univariate analysis results of candidate covariates and total social isolation score (*n* = 337).

Variable	Analytical method	Statistic/effect size	*p*-value	Included in final model
Age (years)	Pearson correlation	*r* = −0.18	0.001	Yes
Sex	Independent samples *t*-test	*t* = −0.87	0.385	Yes
Educational level	Spearman correlation	*ρ* = 0.15	0.005	Yes
Marital status	Independent *t*-test	*t* = 2.45	0.015	Yes
Diabetes duration (years)	Spearman correlation	*ρ* = −0.11	0.042	Yes
CHD duration (years)	Spearman correlation	*ρ* = −0.09	0.095	Yes
Number of comorbidities	Spearman correlation	*ρ* = −0.16	0.003	Yes
HbA1c (%)	Pearson correlation	*r* = −0.08	0.138	Yes

#### 3.7.2 Definition of the final model and multicollinearity assessment

The “fully adjusted model” in this report refers specifically to the multiple linear regression model that simultaneously included the primary independent variable (latent classes of symptom experience) and all pre-specified covariates. No variables were excluded from this final model based on univariate test results, collinearity, missing data, or model fit statistics. To formally assess multicollinearity, variance inflation factor (VIF) values were calculated for all independent variables. To assess multicollinearity, we calculated the variance inflation factor (VIF), and all the VIF values of the variables were below 2 (range: 1.05–1.82), indicating that the collinearity problem was not severe.

#### 3.7.3 Multivariate regression results and model fit

**Figure 3:**
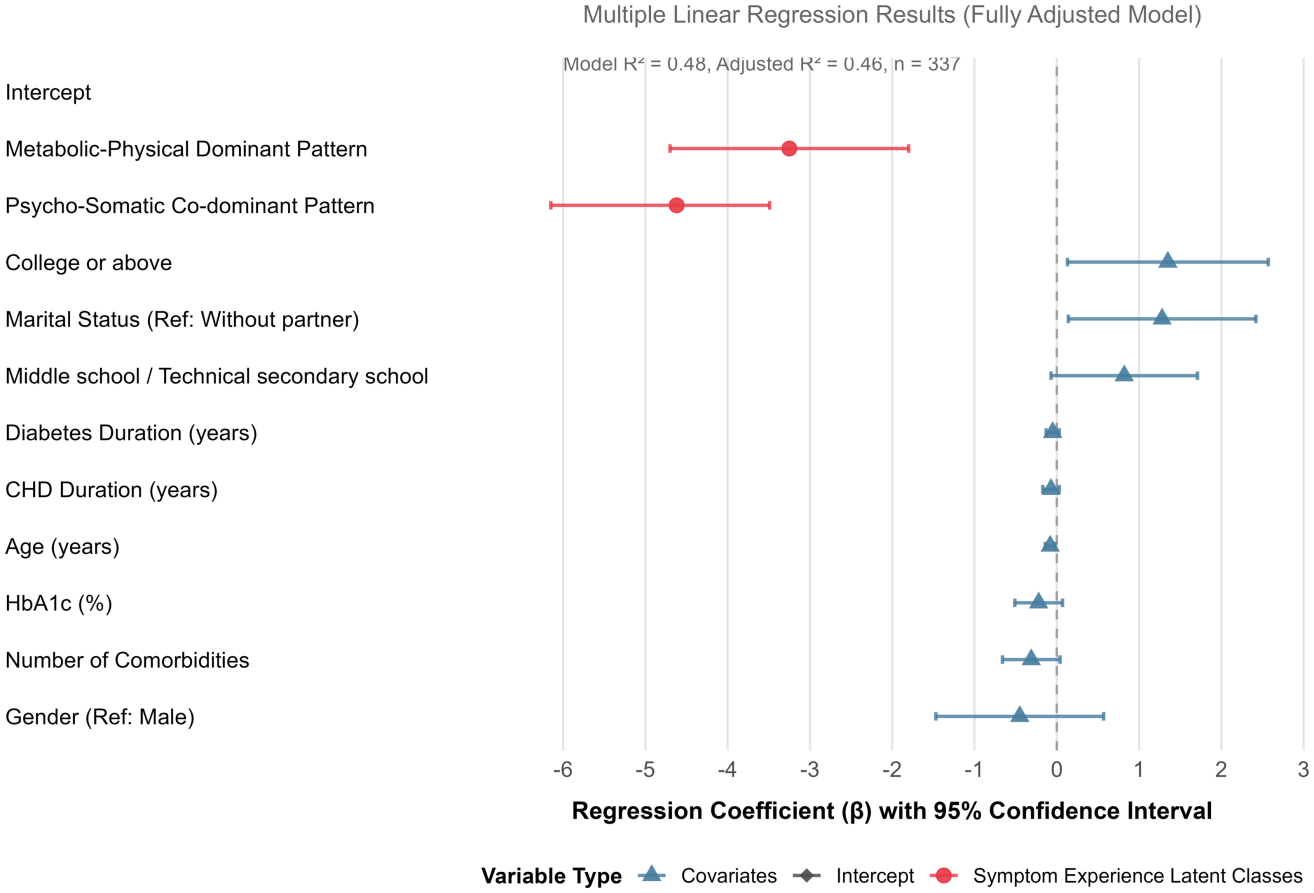
Forest plot of multiple linear regression of potential categories of symptom experience and social isolation.

The fully adjusted multiple linear regression model was statistically significant (*F* = 22.35, *p* < 0.001) and explained a substantial proportion of variance in social isolation scores (*R*^2^ = 0.48, Adjusted *R*^2^ = 0.46). Detailed results are presented in Table 10. After adjusting for all covariates, a significant independent association was found between symptom experience latent classes and social isolation. Specifically, compared to patients in Class 1 (Low Burden-Balanced Pattern), those in Class 2 (Psycho-Somatic Co-dominant Pattern) had significantly lower LSNS-6 scores (*β* = −4.62, p < 0.001). Patients in Class 3 (Metabolic-Physical Dominant Pattern) also showed significantly lower scores than Class 1 (*β* = −3.25, *p* < 0.001), though the magnitude of this association was smaller than that for Class 2. Among the covariates, older age was significantly associated with lower LSNS-6 scores (*β* = −0.08, *p* = 0.008). Higher educational attainment (College or above: *β* = 1.35, *p* = 0.030) and having a partner (*β* = 1.28, *p* = 0.028) were associated with higher LSNS-6 scores. Sex, disease durations, number of comorbidities, and HbA1c level were not statistically significant independent predictors in the final model. The detailed regression results are shown in Table 10, and the main associations are visualized in Figure 3.

**Table 10:** Multiple linear regression analysis of symptom experience latent classes and total social isolation score (fully adjusted model) (*n* = 337).

Variable	*β*	Std. error	Standardized *β*	*t* value	*p-*value	95% confidence interval
Constant	22.15	1.23	—	18.01	<0.001	(19.73, 24.57)
Latent class (ref: Class 1)						
Class 2: Psycho-Somatic Co-dominant Pattern	−4.62	0.68	−0.42	−7.09	<0.001	(−6.15, −3.49)
Class 3: Metabolic-Physical Dominant Pattern	−3.25	0.74	−0.26	−4.39	<0.001	(−4.70, −1.80)
Age (years)	−0.08	0.03	−0.12	−2.67	0.008	(−0.14, −0.02)
Gender (ref: male)	−0.45	0.52	−0.04	−0.87	0.386	(−1.47, 0.57)
Educational level (ref: primary school or below)						
Middle school/technical secondary school	0.82	0.45	0.08	1.82	0.070	(−0.07, 1.71)
College or above	1.35	0.62	0.10	2.18	0.030	(0.13, 2.57)
Marital status (ref: without partner)	1.28	0.58	0.10	2.21	0.028	(0.14, 2.42)
Diabetes duration (years)	−0.05	0.04	−0.06	−1.25	0.212	(−0.13, 0.03)
CHD duration (years)	−0.07	0.05	−0.07	−1.40	0.162	(−0.17, 0.03)
Number of comorbidities	−0.31	0.18	−0.08	−1.72	0.086	(−0.66, 0.04)
HbA1c (%)	−0.22	0.15	−0.06	−1.47	0.143	(−0.51, 0.07)

*β* = Unstandardized regression coefficient; standardized *β* = Standardized regression coefficient. This model is a fully adjusted model. All covariates (including age, gender, educational level, marital status, duration of diabetes, duration of coronary heart disease, number of comorbidities, and HbA1c) were pre-determined based on literature review and clinical significance and included in the model, not selected based on the results of univariate analysis. The model *R*^2^ = 0.48, adjusted *R*^2^ = 0.46, *F* = 22.35, *p* < 0.001.

## 4 Discussion

This study focuses on examining the specific pathway of the “association between symptom experience and social isolation.” The rationale for adopting this research perspective is based on the following considerations. First, from a clinical assessment standpoint, symptoms represent key indicators presented by patients during consultations, while social isolation is a noteworthy psychosocial state. Identifying specific symptom patterns can aid in recognizing groups at higher risk for social isolation. Second, from an intervention feasibility perspective, identifying high-risk populations based on clinically assessable symptoms facilitates the development of an integrated “symptom management-social function maintenance” intervention strategy. Therefore, this study sets social isolation as the dependent variable, aiming to explore the critical issue of using symptom manifestations to identify high-risk populations for social isolation. Building on this, our research is the first to integrate symptom cluster analysis and a person-centered latent profile analysis approach to deeply investigate the heterogeneity of symptom experience in older adult patients with comorbid diabetes and coronary heart disease and to analyze the association between different symptom experience subtypes and social isolation. The main findings confirm the existence of three distinct symptom experience subtypes within this patient population, with the subtype characterized by elevated physical-psychological burden showing the highest degree of association with social isolation. Our findings provide empirical support for the clinical observation of an “association between symptom management and social isolation,” and future longitudinal studies will help further clarify the temporal relationship and potential bidirectional effects between them.

This study yielded several key findings. First, three stable symptom clusters were identified through exploratory factor analysis. While partially consistent with findings from prior research in patients with single chronic conditions, this highlights the complexity and interactions of symptoms in comorbid patients (24). Using latent profile analysis, this study classified patients into three significantly distinct subtypes: the Low Burden-Balanced Pattern, the Psycho-Somatic Co-dominant Pattern, and the Metabolic-Physical Dominant Pattern. This classification extends beyond traditional methods focused solely on symptom counts by revealing qualitative differences in symptom combination patterns. Finally, after controlling for multiple confounding factors, the symptom experience subtypes were independently associated with social isolation. Class 1 (Low Burden-Balanced Pattern) was the largest subgroup (45.4%). Patients in this class had low scores across all symptom clusters, indicating a relatively mild disease burden and more stable clinical condition. This well-managed symptom state may be attributed to effective treatment regimens, stronger personal self-management capabilities (such as medication adherence, healthy diet, and exercise) (25), or the presence of a more robust social support system, thereby establishing a positive cycle of symptom management and quality of life (26). The identification of this subgroup holds significant positive implications, suggesting to clinicians that a considerable proportion of comorbid patients can achieve good health status through optimized management. Their successful experiences warrant further exploration and dissemination.

Second, Class 2 (Psycho-Somatic Co-dominant Pattern) represents the most clinically complex subgroup (31.8%) and demonstrates the strongest association with social isolation. Patients in this class exhibit a high burden on both the “Cardiorespiratory-Fatigue Symptom Cluster” and the “emotional-perceptual symptom Cluster.” This phenomenon of concurrent physical and psychological symptoms aligns closely with the core concept of the “Symptom Synergy Model” (27, 28), which posits that physical discomfort and psychological distress do not exist in isolation but are likely interrelated, potentially forming a vicious cycle where “physical symptoms lead to psychological distress, which in turn amplifies the perception of physical symptoms” (29). This dynamic may explain why this subgroup experiences a heavier overall burden, and why their functional status and quality of life warrant particular attention. Third, the characteristics of Class 3 (Metabolic-Physical Dominant Pattern) (22.8%) differ. The symptom burden in this class is primarily concentrated in the “Metabolic-Related Symptom Cluster” and the “Cardiorespiratory-Fatigue Symptom Cluster.” This pattern suggests that for a subset of patients, the primary challenge stems from the pathophysiological processes of the diseases themselves—namely, the core symptoms arising from disordered glucose metabolism and associated autonomic dysfunction, which consequently trigger significant physical fatigue (30). Compared to Class 2, their emotional-perceptual symptoms are less prominent, possibly reflecting a different underlying disease-driving mechanism. This also implies that intensified management targeting metabolic issues may yield greater benefits for these patients. Our findings resonate with the recent research trend focusing on heterogeneity among patients with comorbidities. For instance, Smith et al. (31) identified a similar “high psycho-somatic burden” subtype in patients with heart failure, which was associated with significantly higher rates of rehospitalization and mortality. By successfully applying this advanced classification paradigm to the comorbid population of diabetes and coronary heart disease—a group of significant public health importance—this study further validates the substantial value of symptom experience-based latent profile analysis for precisely identifying high-risk subgroups.

A key finding of this study is the significant association between different symptom experience subtypes and social isolation, with patients of the Psycho-Somatic Co-dominant Pattern (Class 2) exhibiting a far greater risk of social isolation than other classes. The underlying mechanisms of this association are multi-pathway and multi-level, which can be primarily explained through the following pathways. First is the functional limitation pathway, representing the most direct physiological mechanism (32, 33). Severe physical symptoms, such as persistent fatigue, shortness of breath, and pain, are directly associated with a significant decline in patients’ physical activity capacity. This likely affects their ability to engage in basic activities requiring physical effort, such as going out or participating in social gatherings, thereby linking to reduced social participation. Next is the psychological-behavioral pathway. The substantial psychological burden characteristic of Class 2 patients—including persistent tension, irritability, and sadness—is typically associated with their social motivation and behavior. This internal psychological distress can lead to diminished interest in socializing, reduced energy, and may be accompanied by a degree of stigma, correlating with patient behaviors that involve avoiding social interactions (34, 35). It is particularly important to emphasize that when physical symptoms and psychological burden coexist, they may produce an amplifying “1 + 1 > 2” effect (36, 37). Physical discomfort exacerbates psychological distress, while psychological distress, in turn, reduces patients’ willingness and ability to cope with and manage physical symptoms, collectively pushing patients toward deeper social isolation. Disease management burden pathway: Complex symptom clusters imply more burdensome and time-consuming disease management tasks (38, 39). For instance, frequent blood glucose monitoring, taking multiple medications, and managing episodes of polyuria or hypoglycemia consume a considerable amount of the patient’s time and energy. This high-intensity “illness work” itself may encroach upon time and energy originally allocated for social activities, leisure, and family life, leaving patients neither the inclination nor the capacity to maintain normal social connections. A crucial finding is that while the degree of social isolation in patients with the Metabolic-Physical Dominant Pattern (Class 3) is higher than in Class 1, it is significantly lower than in the Psycho-Somatic Co-dominant Pattern (Class 2). This contrast suggests that, in relation to social isolation, the co-occurrence of psychological symptoms may be a feature requiring greater focus. This indicates that social isolation is not merely associated with declining physical function but is also closely linked to the patient’s psychological experience. Therefore, in clinical interventions, focusing solely on alleviating physical symptoms while neglecting the identification and management of psychological distress may inadequately address the issue of social isolation. Integrating psychosocial support with physical disease management may be an important direction to consider.

The findings of this study carry clear clinical implications. By identifying the two distinct symptom experience subtypes—the “Psychosomatic Co-occurring Subtype” and the “Metabolic-Somatic Dominant Subtype”—clear intervention targets are provided for clinicians. To translate these findings into practice, we recommend establishing an integrated screening and intervention pathway within clinical settings. First, rapid symptom screening for older adult patients with diabetes and coronary heart disease can be implemented in outpatient clinics, focusing on core symptoms such as fatigue, shortness of breath, emotional distress, hunger, and polyuria. This enables initial assessment of their symptom experience pattern and identification of high-risk subtypes. Building on this, for patients identified with the “Psychosomatic Co-occurring Subtype,” the intervention focus should prioritize implementing a collaborative care model involving endocrinology, cardiology, and psychiatry/psychology departments. This approach integrates the management of physical symptoms with cognitive-behavioral therapy to simultaneously alleviate both physiological discomfort and psychological distress. For patients with the “Metabolic-Somatic Dominant Subtype,” efforts should be directed towards enhancing pharmaceutical care to optimize blood glucose control, developing individualized exercise prescriptions, and strengthening the monitoring and management of their metabolism-related symptoms. Critically, for all identified high-risk patients, especially those with a heavy symptom burden, social network assessment should be incorporated into routine care. Proactive efforts should be made to link them with social support resources. This approach helps construct a comprehensive intervention model that spans from symptom identification to psychosocial support, ultimately facilitating a shift from disease management to whole-person care. Future research could explore the following directions: 1) Conducting longitudinal studies to clarify the causal and temporal relationships between symptom experience subtypes and social isolation; 2) Designing and evaluating the effectiveness of personalized management programs tailored to different symptom experience subtypes; 3) Investigating potential moderating or mediating variables that influence the relationship between symptom experience subtypes and social isolation.

Although social isolation and loneliness often co-occur, they differ in their nature, influencing factors, and health consequences. Social isolation is more readily measured through objective indicators (e.g., frequency of social contact, network size) and is strongly associated with endpoint events such as mortality and cardiovascular incidents (40). In contrast, loneliness is a highly subjective experience; even individuals with active social lives may feel profoundly lonely (41). This study focuses on social isolation primarily based on the following considerations: within the context of chronic disease management, objective social network support is an observable, assessable, and potentially more actionable intervention target at the community level (42). Particularly for older adults with comorbidities, identifying high-risk populations whose objective social connections are disrupted due to a heavy symptom burden holds direct priority significance for public health interventions. However, we fully acknowledge that incorporating an assessment of loneliness would provide a more comprehensive understanding of the complete impact pathway of symptom burden on patients’ psychosocial health.

This study has several limitations. First, the cross-sectional design precludes inferring causal relationships between symptom experience subtypes and social isolation. Regarding the external validity of this study, important considerations must be noted. The convenience sampling method employed, with all participants recruited from a single tertiary hospital, limits the generalizability of the findings to broader populations. Specifically, the sample may not fully represent older adults with diabetes and coronary heart disease who receive care in community healthcare settings, primary care centers, or those not engaged with the healthcare system. Furthermore, China’s unique healthcare system, cultural context, social and family structures, and perceptions of chronic disease management may differ significantly from those in other countries or regions. These factors could influence symptom presentation, reporting styles, and the patterns of association with social isolation. Finally, the assessment of both symptoms and social isolation relied on patient self-report, which is susceptible to recall bias. From a methodological perspective, this study utilized principal component analysis (PCA) for exploratory factor analysis to identify symptom clusters. It is acknowledged that PCA is primarily a data reduction technique, whereas common factor analysis methods (e.g., principal axis factoring) are theoretically more aligned with models of “latent constructs.” The choice of PCA was based on its practicality for initial exploration, its goal of maximizing explained variance, and its widespread use in clinical research. We acknowledge this methodological distinction, and future studies could employ more sophisticated methods, such as confirmatory factor analysis or models combining factor analysis, for validation.

## 5 Conclusion

This study identified three distinct latent classes of symptom experience based on symptom clusters among older adults with comorbid diabetes and coronary heart disease, and revealed an independent association between these classes and social isolation. Specifically, the Psycho-Somatic Co-dominant Pattern showed the strongest association with social isolation, followed by the Metabolic-Physical Dominant Pattern, whereas the Low Burden-Balanced Pattern was associated with the least social isolation. These findings highlight the importance of a person-centered approach in clinical practice. By recognizing the heterogeneity in symptom experiences, clinicians can better identify subgroups with different profiles. For patients exhibiting the psycho-somatic pattern, integrated management strategies that address both physical and psychological dimensions, alongside social support considerations, may be particularly relevant. Such tailored approaches could support more individualized care aimed at improving overall wellbeing in this comorbid population.

## Data Availability

The original contributions presented in the study are included in the article/supplementary material, further inquiries can be directed to the corresponding author.
